# Fostering positive attitudes toward food in individuals with restrained eating: the impact of flexible food-related inhibition

**DOI:** 10.1186/s40337-024-00995-0

**Published:** 2024-03-20

**Authors:** Shir Berebbi, Hadar Naftalovich, Noam Weinbach, Eyal Kalanthroff

**Affiliations:** 1 Department of Psychology, The Hebrew University of Jerusalem, Jerusalem, Israel; 2 School of Psychological Sciences, University of Haifa, Haifa, Israel; 3 Department of Psychiatry, Columbia University Medical Center, New York, USA

**Keywords:** Individuals with restrained eating, Inhibition, Inhibition training, Cognitive flexibility, Implicit association test, Stop-signal task

## Abstract

Individuals exhibiting restrained eating behaviors demonstrate increased inhibitory control when exposed to food-related stimuli, indicating the presence of an automatic food-inhibition association. Existing literature proposes that this association contributes to the devaluation of food within this population. Efforts to disrupt this association by promoting the complete elimination of the inhibition of food responses have resulted in increased food consumption but have also led to heightened food-related anxiety in individuals with restrained eating behaviors. In the current investigation, we investigated whether a novel *flexible* food response/inhibition computerized task could yield favorable changes in attitudes toward food in individuals with restrained eating. We randomly assigned 78 females who engage in restrained eating to one of three training groups. In the flexible response/inhibition group, participants were instructed to equally inhibit or respond to food stimuli. In the response group, participants consistently responded to food stimuli, while in the inhibition group, participants consistently inhibited their response to food cues. Implicit attitudes toward food were assessed both before and after the manipulation. To examine the stability of the effect of the training, participants also engaged in a seemingly unrelated bogus taste test. Our results revealed that only the flexible response/inhibition group demonstrated a significant improvement in positive attitudes toward high-calorie foods after eating, while there were no observable changes in negative attitudes among the other two groups. These findings suggest that promoting a balance between the responding and inhibiting responses to food stimuli can increase positive attitudes toward food amongst individuals with restrained eating.

**Supplementary Information:**

The online version contains supplementary material available at 10.1186/s40337-024-00995-0.

## Abstract (plain language summary)

Restrained eaters are individuals who chronically avoid eating to reduce their weight. Researchers have found that these individuals also hold negative attitudes toward food. This may be because of the association created between food and inhibition, our ability to stop an urge or behavior. Researchers attempted to promote healthy eating by training individuals to associate food with disinhibition. This resulted in higher food consumption but it also increased negative attitudes toward food in those individuals. We propose that this is because disinhibition of food is not consistent with the internal goals of these individuals. The current study tested whether training individuals who restrict their eating to respond more flexibly to food would increase positive attitudes toward food. Therefore, we recruited 78 females who restrict their eating into three groups: one where participants were asked to either respond or inhibit their response to images of food in a computerized task, one where participants were asked to *respond* to all food images, and one where participants were asked to *inhibit* their response to all food images. We found that only the flexible group showed more positive attitudes toward food. There were no noticeable changes in the attitudes of the other two groups. These findings suggest that encouraging flexible responses/inhibition to food enhances positive attitudes toward food in individuals who restrict their eating.

**Supplementary Information:**

The online version contains supplementary material available at 10.1186/s40337-024-00995-0.

## Background

Individuals with restrained eating employ restrictive diets to control their weight [26]. These diets have been associated with increased levels of depression, stress, abnormal attitudes toward weight and eating, and low self-esteem [20]. Notably, restrained eating reflects individuals' cognitive and behavioral efforts to manage their weight [39] and does not necessarily lead to lower BMI [16, 27]. To maintain their restrictive diets, individuals with restrained eating often rely on strict and rigid rules regarding their food choices and meal timings [26]. Adhering to such rules requires the active use of inhibitory control, namely, the ability to suppress or delay unwanted, irrelevant, or distracting behaviors, thoughts, or emotions [3, 9, 12, 21, 25, 26]. The connection between food and inhibition in restrained eaters has been demonstrated in several studies, indicating that food-related cues trigger inhibition in this population [12, 43]. Simultaneously, food-related cues also trigger explicit negative attitudes in these individuals, such that restrained eaters exhibit heightened evaluations of the negative aspects of high-calorie foods [32, 33]. Emerging evidence suggests a robust interconnection between these seemingly disparate phenomena, namely inhibitory control and food-related attitudes. For example, researchers have demonstrated that intense execution of inhibition toward a stimulus can result in a less positive attitude toward that stimulus [7, 11, 44]. Hence, the goal of the current study is to investigate whether disassociating food-related cues and inhibition, using a computerized task, can reduce the negative association of food cues in restrained eaters.

The association between food and inhibition has been the subject of numerous investigations. The term food-inhibition association [23] refers to the effect of exposure to food stimuli on inhibitory abilities. In the general population, exposure to food stimuli, generally leads to reduced inhibition (i.e., more food consumption) [12, 13, 29] and increased food cravings [31] when compared to exposure to neutral non-food stimuli. Some researchers have discussed this finding in light of the evolutionary importance of eating, which led to researchers suggesting that there are specific neuronal and behavioral mechanisms meant to ensure appropriate food consumption [8, 37, 40]. Interestingly, individuals with restrained eating have shown an increased level of inhibition when presented with high-calorie foods, compared to their baseline inhibition [12], a finding that has also been replicated in patients with anorexia nervosa [49]. This intriguing effect, which can be viewed as a 'reversal' of evolutionary processes, has been suggested to play a crucial role in the development and maintenance of disordered eating [17]. Specifically, it has been proposed that years of practicing inhibition in response to food stimuli can lead to the formation of a strong food-inhibition association in individuals with disordered eating [50]. This food-inhibition association can significantly influence the attitudes toward food in these individuals and may lead to the automatic inhibition of eating behaviors in response to food stimuli, thereby maintaining restrained eating behaviors.

The relationship between inhibition and attitudes/emotions is a complex and dynamic one [38]. Repeated inhibition targeted at a specific stimulus can exert profound effects on attitudes and emotions associated with said stimulus [11, 44]. This phenomenon can be explained through the Behavior Stimulus Interaction (BSI) model [44]. According to this framework, when a stimulus elicits an approach reaction, but environmental cues indicate that approaching the stimulus is unfavorable (and, thus, the approach behavior should be inhibited), a dynamic interplay emerges between the stimulus and behavioral inhibition, resulting in the modification of attitudes toward the stimulus. Veling et al. [44] demonstrated that the repetitive activation of inhibition toward positive stimuli leads to their devaluation. For example, imagine an individual who loves dogs and then gets bitten by one. According to the BSI model, if that individual will then consistently suppress or inhibit their responses to this particular stimulus (dogs), then over time, the individual will become increasingly sensitized to the stimulus, and develop negative attitudes and emotional reactions toward the stimulus. Consistent with this model, research shows that when food, which commonly serves as a positive stimulus that evokes approach behavior, is linked to inhibition, individuals will rate those foods as less liked [7, 45]. For example, Chen et al. [6] showed that associating specific foods with inhibition (by asking participants not to respond to trials of a cognitive task containing these foods) leads to the devaluation of these food stimuli (see also: Houben [19]). Hence, in the case of individuals with restrained eating, the persistent and repetitive inhibition toward food may potentially amplify pre-existing negative attitudes toward food, perpetuating a vicious cycle of both negative attitudes toward food and disordered eating behaviors.

Several therapeutic interventions have focused on the association between food and inhibition. Specifically, in recent years, researchers have explored the use of cognitive tasks to manipulate the food-inhibition relationship to reduce clinical symptoms [5, 28, 41]. One common approach to modulate this relationship is by utilizing a cognitive task that requires inhibitory control, such as the stop-signal task [24, 47]. While originally designed to assess inhibitory control, researchers have employed this task, and similar ones, to create a new 'learned' reflexes by associating target stimuli with inhibition [46]. For instance, Hochman et al. [18] manipulated the proportion of specific trials that necessitated inhibition. This was done by ensuring that a high proportion of the conditioned stimulus (e.g., a green circle) was consistently followed by a stop-signal, which indicated to the participant the need for inhibition. Meanwhile, the unconditioned stimulus (e.g., a blue rectangle) was rarely followed by a stop-signal. Through this approach, researchers were able to condition levels of automatic inhibition triggered by the conditioned stimulus (see also: Manasse et al. [28]). In the context of eating behaviors, researchers have utilized similar tasks to manipulate the automatic association between food cues and inhibition. In studies involving healthy controls, training tasks were employed to condition different types of food with either inhibition or response, revealing that participants subsequently exhibited a preference for food associated with response over food associated with inhibition [1, 30, 45]. In addition, overweight and obese participants who underwent training associating high-calorie snacks with inhibition displayed a decrease in their liking of these snacks and these participants also lost more weight over time [22]. Thus, taken together with the above research, it is evident that the food-inhibition relationship is not only central to disordered eating but can also be manipulated using computerized tasks.

Until recently, researchers focused on creating a food-inhibition association to examine how such an association can facilitate weight loss. The question then arises whether the food-inhibition association can also be manipulated to decrease the inhibitory response to food stimuli, which would help individuals with restrained eating return to healthier approach attitudes toward food. Weinbach et al. [48] employed a similar training approach with individuals with restrained eating, this time focusing on establishing an association between food and response/disinhibition. Participants completed a modified version of the stop-signal task, wherein neutral images (e.g., table, chair) were frequently followed by a stop cue, while food-related images were never accompanied by such a stop cue. The results of this study indicated that following this training procedure, individuals with restrained eating were more likely to increase their food consumption in an unrelated bogus taste test. Interestingly, individuals with restrained eating who underwent the food-response training also reported higher food-related anxiety after the task, compared with their pre-training food-related anxiety levels. This study also included another group of restrained eaters who underwent similar inhibitory training, but without a specific association between food and response/inhibition, as stop-signals appeared equally in both food and non-food trials. This group, which was initially a control group, exhibited more positive attitudes toward food and even a reduction in their food-related anxiety [48]. The researchers suggested that this may be because this condition allowed for a more flexible approach to food, enabling the participants to alternate between response inhibition and response execution when exposed to food stimuli. However, it is important to note that a food-inhibition group was not included in that study, which limits our ability to draw definite conclusions about the impact of these training procedures on attitudes towards food, especially in the case of individuals with restrained eating.

The literature reviewed above provides evidence that different levels of the food-inhibition association can lead to the devaluation of food cues and, perhaps, can even increase food-related anxiety. Consequently, if we want to prevent the devaluation of food, and perhaps even to improve attitudes toward food, it is imperative to explore strategies that can effectively counter food-related anxiety and foster positive attitudes toward food. Previous studies suggest that utilizing a training procedure aimed at enhancing inhibitory *flexibility* can lead to a positive emotional evaluation of food, which can persist even after eating, among individuals with restrained eating [48]. Such training seeks to establish a balance between inhibiting and responding to food cues. Therefore, the goal of the current study was to investigate whether an inhibitory task designed to enhance flexible inhibition toward food would result in more positive attitudes toward food, especially compared to both food-response and food-inhibition training groups.

To achieve this aim, the experiment included three groups, each performing a different version of the food stop-signal task: The first group, referred to as the *flexible response/inhibition group*, completed the task in which food stimuli were equally associated with both responses and inhibition. The second and third groups, named the *food-response* and *food-inhibition* groups, completed the task in which food stimuli were consistently associated with either response or inhibition, respectively. Notably, the task consisted of only one brief training session. Pre- and post-training implicit association tests were used to measure implicit attitudes toward food. To ensure that the effect of the training persists even after food consumption, participants were also asked to eat as part of a seemingly unrelated Bogus taste test. Based on the literature reviewed earlier, we hypothesized that the flexible response/inhibition group would exhibit more positive attitudes towards high-calorie foods after completing the food stop-signal task, compared to both other groups. Furthermore, we anticipated that this improvement would persist even after eating. Additionally, if the brief (single session) training affects eating, we expected that food consumption would be highest in the food-response group, lowest in the food-inhibition group, and intermediate in the flexible response/inhibition group.

## Methods

### Participants

Ninety-one female individuals with restrained eating participated in the study in return for a small monetary reward (~ 12 USD) or course credit. Because restrained eating to control weight is far more common in females than in males [36], only female individuals with restrained eating were recruited for the current study. The demographics and clinical characteristics of the sample are presented in Table 1. All participants had normal or corrected-to-normal vision, and all were naïve as to the purposes of the experiment. Participants were recruited from an undergraduate university sample. Potential participants were initially screened using an online survey, which included 4 questions on a 1–5 scale, from the Restrained Eating subscale of the Dutch Eating Behavior Questionnaire (DEBQ-R; van Strien et al. [42]). Participants with a mean score of above 3 on these 4 items were invited to take part in the study. Upon arriving at the lab, participants completed the full DEBQ-R, and only participants with a mean score of above 3 were included in the study [51]. Finally, thirteen participants were excluded from the analyses for being outliers (> 3 SD from the group’s mean) in reaction time (RT) and accuracy rates in the first or second Implicit Association Test (IAT). The final sample included 78 female participants. The participants were randomly assigned to either the *flexible inhibition, the food-response,* or the *food-inhibition* group.

**Table 1:** Characteristics of the sample in the three experimental groups

	Flexible inhibition n = 26	Food-response n = 26	Food-inhibition n = 26	*F*	*p*-value
Age	23.65 (5.00) [18.00–45.00]	22.92 (2.78) [18.00–33.00]	23.12 (1.86) [19.00–27.00]	*F*(2,75) = 0.31	.74
BMI	23.36 (4.59) [17.95–36.71]	24.68 (4.84) [17.96–37.46]	22.07 (2.82) [15.64–28.88]	*F*(2,75) = 2.53	.09
DEBQ-restrained	4.07 (0.57) [3.10–4.90]	3.98 (0.56) [3.00–5.00]	3.73 (0.47) [3.00–4.80]	*F*(2,75) = 2.75	.07
DEBQ-emotional	2.94 (1.11) [1.31–5.00]	2.98 (1.00) [1.31–5.00]	3.09 (1.19) [1.23–5.00]	*F*(2,75) = 0.14	.87
DEBQ-external	3.54 (0.69) [1.67–4.50]	3.78 (0.55) [2.50–4.70]	3.53 (0.75) [2.10–4.90]	*F*(2,75) = 1.18	.31
DASS-21 depression	27.54 (8.10) [14.00–44.00]	29.54 (8.98) [16.00–49.00]	27.73 (9.74) [53.00–14.00]	*F*(2,75) = 0.39	.68
DASS-21 anxiety	28.69 (9.36) [15.00–48.00]	30.92 (9.78) [16.00–47.00]	28.35 (10.30) [14.00–50.00]	*F*(2,75) = 0.53	.59
DASS-21 stress	23.77 (9.00) [13.00–51.00]	24.85 (7.96) [14.00–40.00]	24.92 (10.49) [14.00–49.00]	*F*(2,75) = 0.13	.88
Baseline hunger	4.00 (2.14) [0.00–7.00]	3.31 (2.75) [0.00–8.00]	4.23 (2.89) [0.00–8.00]	*F*(2,75) = 0.88	.42
Baseline food-related anxiety	5.12 (2.52) [0.00–10.00]	5.08 (2.70) [0.00–9.00]	4.85 (2.71) [0.00–9.00]	*F*(2,75) = 0.08	.92

Mean, (Standard Deviation), [range] of demographics and clinical characteristics of each of the three experimental groups. One-way analyses of variance (ANOVAs) were carried out to compare between-groups differences in age, BMI (Body Mass Index), all three subscales of DEBQ (Dutch Eating Behavior Questionnaire; restrained, emotional, and external), all three subscales of the DASS-21 (Depression Anxiety Stress Scales-21; depression, anxiety, and stress), and baseline levels of hunger and food-related anxiety. As can be seen, there were no differences between the groups in any of these measures

The sample size was pre-registered (ClinicalTrials.gov ID #NCT05553574). A power analysis using G × Power 3.1 [10], based on the effect size reported in a previous study that compared implicit negative and positive attitudes toward high-calorie foods among obese and normal-weight participants (*η*^2^ = 0.76; Roefs and Jansen [35]), indicated that the current sample allowed for examination of group differences in IAT at a power > 95% with a Type 1 error (α < 0.05). Sample characteristics and between-group comparisons of demographic and clinical variables are presented in Table 1.

### Procedure

**Fig. 1:**
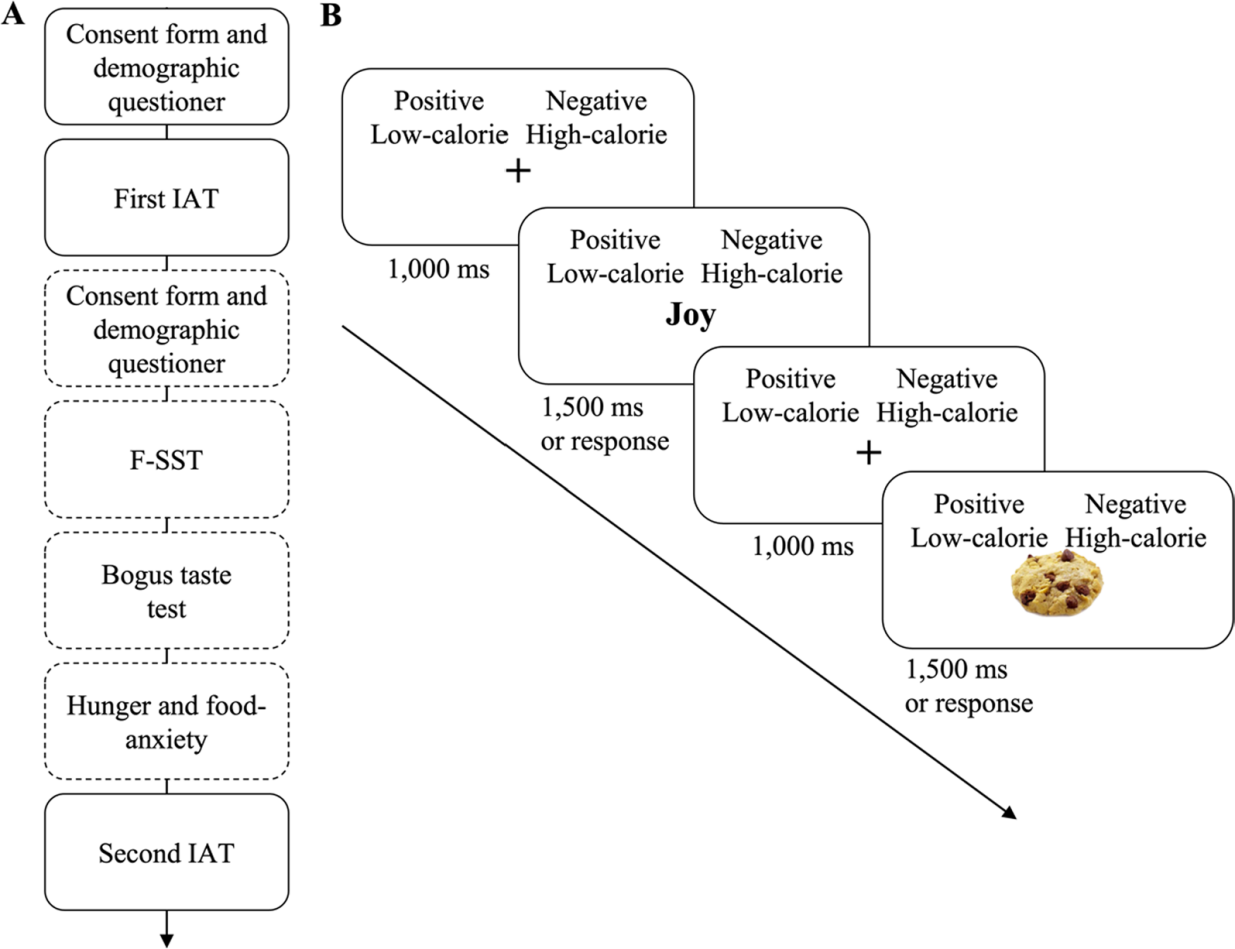
**A** illustrates the course of the experiment. The first and the second Implicit Association Tests (IATs) were presented as one study (continuous line), while food stop-signal task (F-SST) and bogus taste test were presented as a second and separate study (dashed line). Participants were told that they needed to wait 20 min between the first and second IAT, and "study 2" was offered to them to take advantage of this time. **B** illustrates a trial in the IAT that examines the association between high-calorie food and negative words

The study was approved by the Ethics Committee of the Hebrew University of Jerusalem (HUJI-2021–06011) and followed APA ethical standards. The study was preregistered (ClinicalTrials.gov ID #NCT05553574). All participants gave their informed consent before they participated in the study. To reduce differences in a-priori states of hunger, participants were asked to refrain from eating one hour prior to the experiment. To ensure that participants were naïve to the purpose of the experiment, the experiment was presented as two separate studies as elaborated below (see Fig. 1A). First, participants completed a demographic questionnaire, followed by a question about their levels of hunger (“What is your current level of hunger?”) and a question about their state of food-related anxiety (“What is your current level of anxiety that is related to food?”), on which participants were asked to respond using a visual analog scale (VAS). Next, participants completed the first IAT followed by another administration of the two VAS questions about hunger and food-related anxiety. At this point, the experimenter explained that they now must wait 20 min before they can continue with the study and that, if they wish, they can spend this time completing a separate study in the lab, for which they need to sign a separate consent form and will be compensated separately. All the participants chose to participate in the “second” study. Then, participants were randomly assigned to one of the three groups (flexible inhibition, food-response, or food-inhibition), and completed the food stop-signal task (F-SST) according to their group. Following the task, participants completed a bogus taste task to measure food consumption, followed by the two VAS questions about hunger and food-related anxiety. Next, participants were told they can now continue with the original study and completed the second IAT. Finally, participants completed two self-report questionnaires: the DEBQ and the Depression Anxiety Stress Scales-21 (DASS-21; Fig. 1A). The tasks (IAT and F-SST) were administered using OpenSesame 3.3.4, and all the questionnaires were administered via Qualtrics.

### Measures

Implicit association test (IAT; Greenwald et al. [14, 15]; Fig. 1B) is a measure of automatic implicit associations. The IAT used in this study consisted of two main classification tasks (in this case to classify words or to classify images of food), where participants were asked to classify whether the presented stimulus belongs in one of two categories. Performance on these tasks is affected by the strength of the association between the stimulus and the different categories. In the current study, we used two categories, each with two subcategories. The first category was *food* and it contained 10 high and 10 low-calorie food pictures that were selected from the “food pics” database [4]. The second category asked participants to classify whether certain words were pleasant or unpleasant using 10 positive and 10 negative words. The full list of the images and the words can be seen in Additional file 1: Table S1. The test consisted of seven blocks, in which participants were asked to classify the pictures (high vs. low-calorie) or the words (pleasant vs. unpleasant) that were presented on the screen, using the categories that appeared on the screen (Fig. 1B). Each trial started with a 1,000 ms fixation followed by a target stimulus that appeared for 1,500 ms or until keypress. Participants were instructed to respond to the content of the stimuli (high vs. low-calorie food or pleasant vs. unpleasant word) with the “z” key or the "/" key on the keyboard, using the index finger of both hands, as quickly as possible. In the case of an incorrect response, a small red 'X' appears at the bottom of the screen and disappears the moment participants press any key on the keyboard. Each trial ended with a 250 ms blank screen inter-trial interval.

Blocks 1, 2, and 5 consisted of 20 trials of only one category each (blocks 1 and 5 consisted of food stimuli and block 2 consisted of pleasant and unpleasant words). These blocks were considered practice blocks and were not further analyzed. Blocks 3 and 6, which were also considered practice blocks and not further analyzed, consisted of both categories (food and pleasant and unpleasant words) presented together (Fig. 1B), and participants were required to respond to the target stimulus, deciding whether it is a high or low-calorie food picture or a pleasant or unpleased word. Here, the same key (the “z” key or the "/" key) represented two subcategories, one from each category. For example, in block 3, the participants were required to respond with the “/” key for high-calorie food pictures or to pleasant words and to respond with the "z" key for low-calorie food pictures or to unpleasant words. Blocks 4 and 7 were identical to blocks 3 and 6 (respectively) but consisted of 40 trials each and were considered the target blocks, on which all analyses were carried out. To ensure counterbalancing, the order of blocks was arranged such that for half of the participants, the positions of Blocks 1, 3, and 4 were swapped with those of Blocks 5, 6, and 7, respectively. The full sequence of trial blocks can be seen in Additional file 1: Table S2. RTs were measured for the association between every two subcategories that were paired with the same key on the keyboard. For example, if high-calorie food pictures or pleasant words required responses using the “z” key, faster RTs indicated a stronger association between those categories (and vice versa). To assess the participant's positive attitude toward high-calorie food, we subtracted the mean RT of block 4 (pairing high-calorie food images with pleasant words and low-calorie food with unpleasant words) from the mean RT of block 7 (pairing high-calorie food images with unpleasant words and low-calorie food with pleasant words) and divided the outcome by the standard deviation calculated for all trials in blocks 4 and 7 combined [15]. A positive result indicates a stronger association between high-calorie food and pleasant words, as well as between low-calorie food and unpleasant words.

Food stop-signal task (F-SST; adapted from: Ganor-Moscovitz et al. [12]). The task was originally used to measure inhibitory control of food-related images in individuals with restrained eating. The three versions of the F-SST administered in the current study were used to manipulate the association between food and inhibition. Each trial started with a white fixation presented at the center of a black screen for 1,000 ms. Next, an image of either a food or non-food item was presented at the center of the screen (i.e., a go signal). Participants were instructed to press the “y” key on the keyboard for food stimuli or the "v" key for non-food stimuli with the index finger of both hands as fast and as accurate as possible. The go stimulus was presented for 1,500 ms or until keypress. Forty images of high-calorie food (20 sweet and 20 savories) and 40 non-food images (household items) were selected from the “food pics” database [4] and presented in a random order to participants from all groups (note that the pictures were different from those used for the IAT). Following the classic stop-signal task [24], in 25% of the trials, a stop-signal (i.e., a white frame) was presented. Participants were instructed to withhold their response upon seeing the stop-signal. The duration between the appearance of the go and stop-signal, the ‘stop-signal delay’ (SSD), was subjected to a tracking procedure. Initially, the SSD was set at 250 ms, after every successful stop, the SSD was prolonged by 50 ms (making it more difficult to stop) while after every erroneous response to a stop-signal trial, the SSD was shorted by 50 ms. Each trial ended with a black screen inter-trial interval. The proportions of food stop-signal trials (stop-signal trials with a food picture as a go signal) were manipulated to create three groups. In the flexible response/inhibition group, the stop-signal trials were distributed equally across trials, to have 30 no-food stop-signal trials and 30 food stop-signal trials (creating a balanced association between food and response and inhibition), in the food-response group, all 60 stop trials were non-food trials (associating food with response), and in the food-inhibition group, all 60 stop trials were food trials (associating food with inhibition). In the flexible inhibition condition, there were 2 separate tracking procedures, such that the SSDs were updated separately for food-stop trials and for non-food-stop trials. Each trial ended with a 500 ms inter-trial interval. The task started with 32 practice trials that included feedback and were not further analyzed. The experimental task included 240 trials.

The bogus taste test is a measure of food consumption [34]. Three bowls of palatable snacks containing: button-shaped chocolates (M&M’s), pretzel sticks, and hazelnut biscuits (Loacker), were given to the participants. Participants were asked to “taste the snacks from each bowl” (in a consistent order) and rate them on the following 1–10 scales: taste, sweetness, crunchiness, bitterness, and saltiness. No instructions were given regarding the amount of food that the participants needed to taste. The total amount of snacks eaten was recorded.

### Evaluation questionnaires

Dutch Eating Behavior Questionnaire (DEBQ) is a 33-items self-report questionnaire that consists of three subscales—restrained eating (10 items), emotional eating (13 items), and external eating (10 items). Items are scored on a 5-point Likert scale, ranging from "never" to "very often". The DEBQ has satisfactory reliability and validity [42]. The Cronbach's alpha scores of the current study were 0.72, 0.92, and 0.75 for restrained eating, emotional eating, and external eating, respectively.

The Depression Anxiety Stress Scales-21 (DASS-21) is a 21-items self-report questionnaire that measures depression, anxiety, and stress. Items are scored on a 4-point Likert scale, ranging from "does not describe my behavior" to "my behavior most of the time". The DASS-21 has satisfactory reliability and validity [2]. The Cronbach's alpha scores of the current study were 0.89, 0.92, and 0.90 for depression, anxiety, and stress, respectively.

## Results

**Fig. 2:**
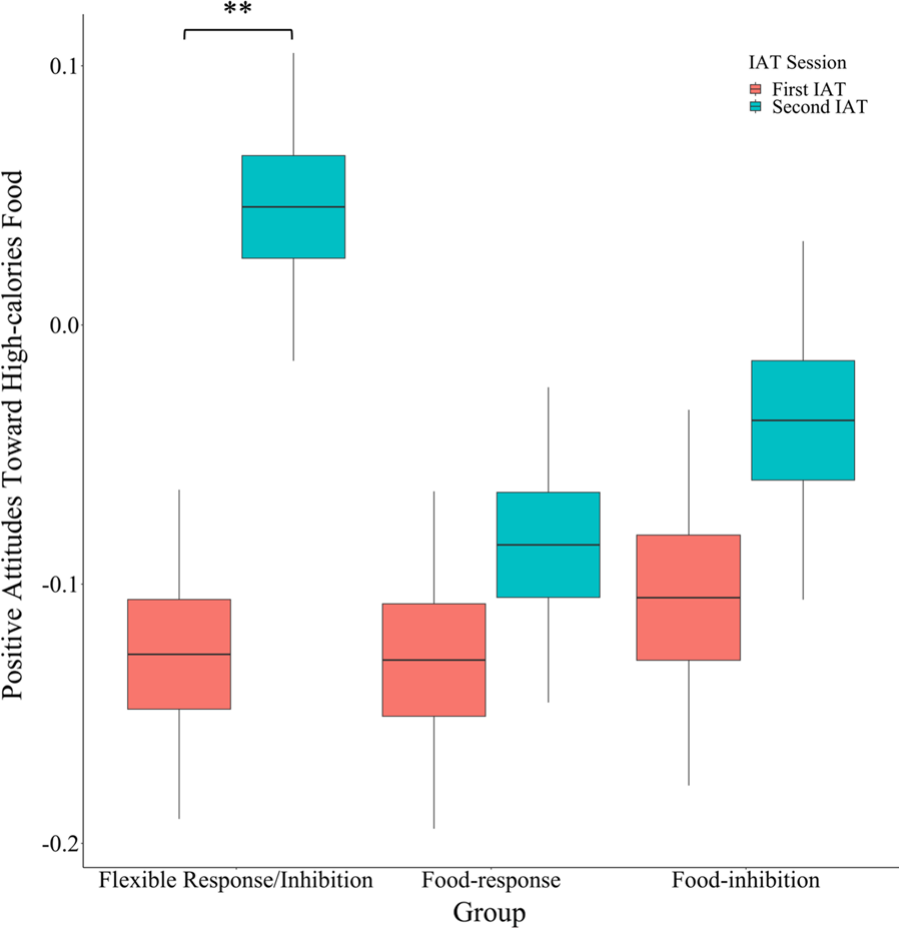
Positive attitudes toward high calories food for the flexible inhibition, the food-response, and the food-inhibition groups, at the Implicit Association Tests (IATs) pre food stop-signal task (F-SST) and post F-SST. The y-axis represents positive attitudes toward high-calorie food and negative attitudes toward low-calorie food. **Significant at p < .01 with Bonferroni correction. Error bars represent one standard error from the mean

The data for this study is available at https://doi.org/10.17632/5hxn8ddwf2.1. As can be seen in Table 1, there were no a-priori significant differences between the three groups in age, BMI, all 3 sub-scales of DEBQ, all 3 sub-scales of the DASS-21, baseline hunger, and baseline food-related anxiety. To test our main hypothesis regarding the effect of the F-SST on attitudes toward food, we carried out a two-way mixed model analysis of variance (ANOVA) on the ‘positive attitudes toward high-calorie food’ data from the IAT task, with a group as a between-subject factor and time (pre vs. post F-SST) as a within-subject factor (Fig. 2). The results yielded no significant main effects for time [*F*(1, 75) = 2.05, *p* = 0.16] nor for group [*F*(2, 75) = 0.09, *p* = 0.91]. The group X time interaction was significant [*F*(2, 75) = 3.19, *p* = 0.05, $\upeta _{p}^{2} = .08$; Fig. 2]. To further investigate this interaction, we conducted planned comparisons on ‘positive attitudes toward high-calorie food’ data, with time as a within-subject factor, for each group separately, using paired samples t-tests, with Bonferroni correction for multiple comparisons. The results indicated a significant difference between the pre and post F-SST only for the flexible response/inhibition group [*t*(75) = 2.87, *p* < 0.01, Cohens' *d* = 0.49], such that scores for the post F-SST IAT were higher (more positive attitudes toward high-calories food and less negative attitudes toward low-calorie food) compared to the pre F-SST IAT. Notably, only in this group, and only after the F-SST, did the attitudes toward food become positive (see Fig. 2; Table 2). There were no significant differences between the pre and post F-SST for the other two groups, (*t*(75) = 0.05, *p* = 0.96 and *t*(75) = 0.44, *p* = 0.66, for food-response and food-inhibition, respectively).

**Table 2:** Results of the Implicit Association Tests (IATs), the food consumption and hunger levels

	Flexible inhibition n = 26	Food-response n = 26	Food-inhibition n = 26
Positive attitudes toward high calories food—First IAT	− 0.13 (0.02)	− 0.13 (0.02)	− 0.11 (0.02)
Positive attitudes toward high calories food—Second IAT	0.05 (0.02)	− 0.08 (0.02)	− 0.04 (0.02)
Food consumption	11.81 (1.72)	14.58 (1.94)	7.92 (0.80)
Baseline hunger	4.00 (0.08)	3.31 (0.11)	4.23 (0.11)
Pre F-SST hunger	3.88 (0.08)	4.00 (0.12)	4.58 (0.11)
Post F-SST hunger	4.38 (0.09)	4.73 (0.10)	4.69 (0.11)

Mean, (one standard error of the mean) data of the three groups of the Implicit Association Test (IAT), the food consumption and hunger levels

**Fig. 3:**
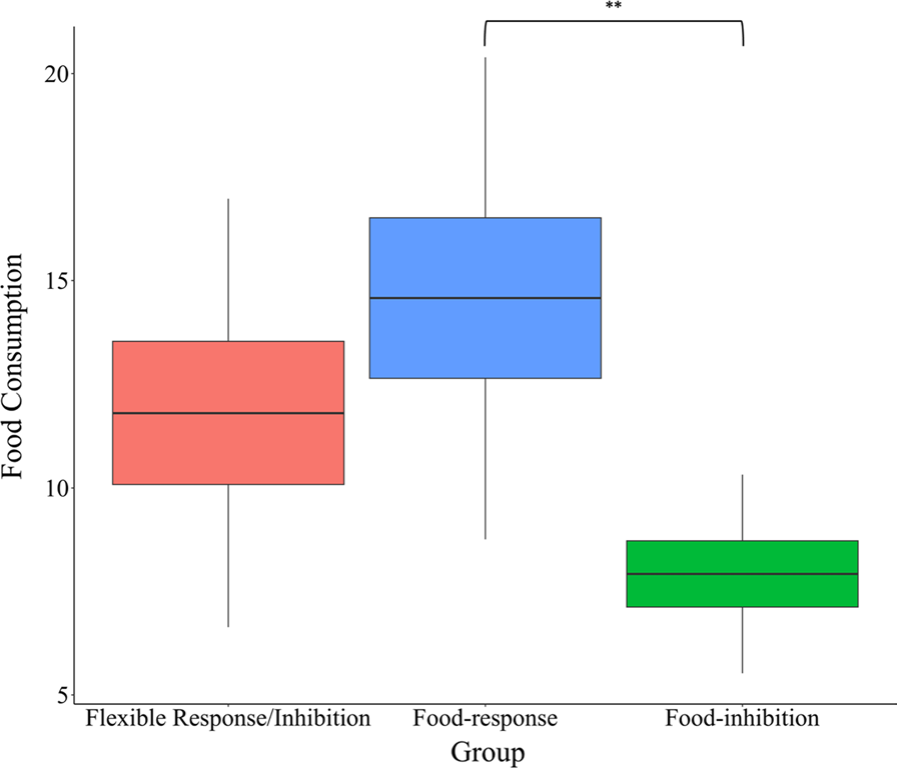
Snack consumption in the flexible inhibition, the food-response, and the food-inhibition groups. The y-axis represents the total number of snacks eaten from the three bowls. **Significant at p < .01 with Bonferroni correction. Error bars represent one standard error from the mean

To examine the difference in food consumption, we calculate the total amount of food consumption (sum of the three snacks) for each participant in each group. We carried out a one-way ANOVA on total food consumption, with a group (flexible inhibition, food-response vs. food-inhibition) as a within-subject factor. The main effect for the group was significant [*F*(2, 75) = 4.55, *p* = 0.01, $\upeta _{p}^{2} = .11$; Fig. 3]. To further investigate this effect, we have conducted planned comparisons between pairs of groups on total food consumption, using independent samples t-tests, with Bonferroni correction for multiple comparisons. The results indicated higher food consumption in the food-response group compared to the food-inhibition group [*t*(75) = 3.00, *p* = 0.01, Cohens' *d* = 0.88]. The difference between the flexible response/inhibition group and the other two groups was not significant [*t*(75) = 1.25, *p* = 0.43; *t*(75) = 1.75, *p* = 0.19, for food-response and food-inhibition groups, respectively; Fig. 3 and Table 2].

To test the differences in hunger during the study, we carried out a two-way mixed model ANOVA on hunger levels, with a group as a between-subject factor and time as a within-subject factor. There was a main effect for time [*F*(1.31, 97.94) = 6.11, *p* < 0.01, $\upeta _{p}^{2} = .08$]. To further investigate this effect, we have conducted planned comparisons using paired samples t-test, with Bonferroni correction. The results indicated that compared to the bassline time point, levels of hunger were significantly higher at the pre F-SST time point [*t*(75) = 2.70, *p* = 0.02, Cohens' *d* = 0.12], and at the post F-SST time point [*t*(75) = 2.91, *p* = 0.01, Cohens' *d* = 0.29]. However, there was no significant difference between the pre F-SST and post F-SST time points [*t*(75) = 1.81, *p* = 0.17]. The main effect for group [*F*(2, 75) = 0.31, *p* = 0.74] and the group X time interaction were not significant [*F*(2.61, 97.94) = 1.40, *p* = 0.25; Table 2].

## Discussion

The current study examined the impact of promoting flexible inhibitory strategies toward food through a novel food-related inhibitory task on implicit attitudes toward high-calorie foods in individuals with restrained eating. In line with our hypothesis, only the flexible response/inhibition group had a significant increase in positive attitudes toward high-calorie foods after completing the food-related inhibitory task (the F-SST) compared to their pre-task attitudes. In contrast, the other two groups did not exhibit any significant changes in attitudes toward food following the training task. Importantly, these results were obtained even after participants consumed food, indicating that the effect of the brief training procedure was not eliminated by actual eating. Additionally, although the differences in food consumption were small, we found that the food-response group consumed more food than the food-inhibition group, which is consistent with Weinbach et al. [48]. As can be seen in Fig. 3, the flexible response/inhibition group fell in between the other two groups, being somewhat closer to the food response group. However, the differences in food consumption between the flexible response/inhibition group and the other two groups were small and non-significant. Importantly, our results revealed no significant differences in hunger levels before and after the F-SST manipulation and no group differences were found. This indicates that our results cannot be directly attributed to food craving or hunger levels. Taken together, these findings suggest that promoting flexible food-related inhibition can enhance positive attitudes towards high-calorie food among individuals with restrained eating, even after eating.

The current study presents empirical evidence demonstrating that a more flexible association between response and inhibition towards food enhances positive attitudes toward high-calorie foods in individuals with restrained eating. This finding is in line with prior research [48], further strengthening the existing knowledge in this area. Previous research has indicated that linking response with food cues can increase eating behavior among individuals with restrained eating [48]. The flexible condition may represent the optimal approach, as it allows for a conflict resolution that ultimately modifies the valence of food, making it less negative or even positive. We propose that promoting both response and inhibition equally facilitates flexible inhibition, enabling a more nuanced perspective on high-calorie foods. In the case of individuals with restrained eating, who exhibit a strong association between food and inhibition compared to their baseline inhibition [12], adopting a more nuanced perspective likely enables them to attenuate the food-inhibition association without perceiving the intervention as completely incongruous with their internal motivations. Hence, the primary contribution of the present study lies in demonstrating that a more flexible training approach can enhance attitudes toward food, perhaps by bypassing the anxiety induced by attempts to convince such individuals to eat.

There is evidence that interventions aimed at increasing the food-response relationship also heighten food-related anxiety and contribute to the devaluation of food in individuals with restrained eating. This outcome is likely attributed to the incongruity between the training’s demand to minimize the inhibition when presented with food and the individual’s automatic motivation to inhibit their urge to eat. In other words, while individuals with restrained eating in the current study may have learned not to restrain themselves in the presence of food cues, such an approach may feel contradictory, ultimately reinforcing their negative attitudes toward food. This finding regarding the effect of inhibiting eating behaviors and attitudes toward eating may also be interpreted in the context of the Behavior Stimulus Interaction (BSI) theory. According to this theory, individuals with restrained symptoms perceive food as a stimulus that elicits avoidance, they’ll develop a negative attitude toward that stimulus. In our study, we show that in the case of food, this model also works in reverse, when an external approach cue is presented to a food stimulus previously associated with avoidance, it can potentially devalue the negative valence associated with the food.

The finding that associating food with flexible inhibition improves attitudes toward food carries significant clinical implications. Firstly, our study demonstrates that promoting healthy eating attitudes and behaviors in individuals with disordered eating relies not only on increasing eating and improving attitudes towards food, but also on fostering flexible inhibition. This insight suggests that interventions targeting flexible inhibition could be beneficial in promoting healthier eating patterns in the long term. Furthermore, our results suggest that utilizing a stop-signal task as training for individuals with restrained symptoms holds potential implications for treatment. Future research should expand the study by introducing longer trainings and investigating whether enhancing flexible inhibition towards food can be effective for individuals with eating disorders.

Moreover, in this study, we examined implicit associations towards food and food consumption immediately after the training. Notably, one important strength of the current study is the fact that participants were required to eat between the first and second implicit association tests. Although the differences in food consumption were relatively small and mostly non-significant, two notable points deserve emphasis. Firstly, the improvement in attitudes toward food observed in the flexible response/inhibition group occurred after eating, indicating the stability and persistence of the task's effect following an actual ecological encounter with food. Secondly, it is noteworthy that the flexible response/inhibition group exhibited enhanced attitudes toward food despite consuming slightly larger quantities compared to the food-inhibition group. Future studies should investigate whether the training effects endure beyond the study's timeframe and whether they are sustained over a longer period, potentially through more intensive training sessions.

## Conclusions

To conclude, the current study utilized a novel food inhibitory task to examine how attitudes toward high-calorie foods change after training an individual to: (a) employ flexible response/inhibition to food cues, (b) respond to food cues, or(c) inhibit their response to food cues, in individuals with restrained eating. As hypothesized, only the flexible response/inhibition group showed increased positive attitudes toward high-calorie food following the intervention. Importantly, this effect was obtained after participants from all groups were asked to eat high-calorie foods. In addition, consistent with previous research, the food-response group demonstrated higher eating levels than the food-inhibition group. The flexible inhibition demonstrated similar levels of eating to the food response, however, the difference between this group and the food inhibition group failed to reach significance. It is plausible that the effects of flexible inhibition training on eating behavior may be more pronounced over a longer duration, and future studies should explore, extend, and sustain training protocols to investigate the potential for greater changes in the emotional valence of food.

### Supplementary Information

**Additional file 1. Table S1:**The images and the pleasant and unpleasant words in the Implicit Association Test (IAT). **Table S2**: The sequence of trial blocks in the Implicit Association Test (IAT).

## Data Availability

The datasets generated and analyzed during the current study are available in the Mendeley Data repository, https://doi.org/10.17632/5hxn8ddwf2.1.

## Abbreviations

ANOVA: Analyses of variance
BMI: Body mass index
BSI: Behavior Stimulus Interaction model
DASS-21: Depression Anxiety Stress Scales-21
DEBQ: Dutch Eating Behavior Questionnaire
DEBQ-R: Restrained Eating subscale of the Dutch Eating Behavior Questionnaire
F-SST: Food Stop-Signal Task
IAT: Implicit Association Test
RT: Reaction time
SSD: Stop-signal delay
VAS: Visual analog scale
