# Supplementary material for: Fostering positive attitudes toward food in individuals with restrained eating: the impact of flexible food-related inhibition
Source: J Eat Disord. 2024 Mar 20;12:41. doi: 10.1186/s40337-024-00995-0 (PMC10956199; doi:10.1186/s40337-024-00995-0)
Supplement: Supplementary file 1 — Additional file 1. Table S1:The images and the pleasant and unpleasant words in the Implicit Association Test (IAT). Table S2: The sequence of trial blocks in the Implicit Association Test (IAT). [file 40337_2024_995_MOESM1_ESM.docx]

**S1.** The images and the pleasant and unpleasant words in the Implicit Association Test (IAT).

| High-calorie food images | Low-calorie food images | Pleasant words | Unpleasant words |
| --- | --- | --- | --- |
| 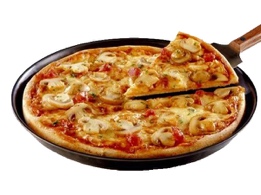 | **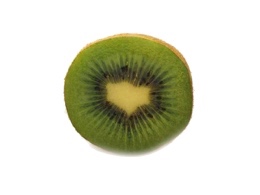** | Love | Crime |
| 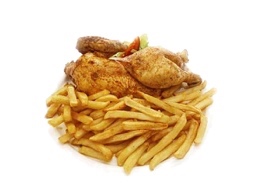 | **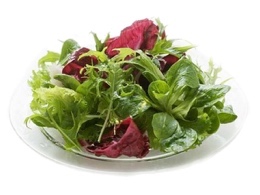** | Smile | Hate |
| 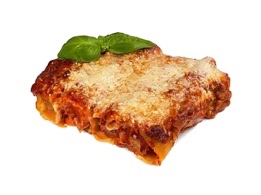 | **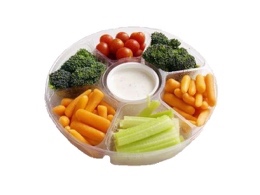** | Kiss | Torture |
| 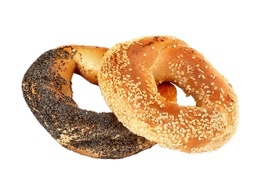 | **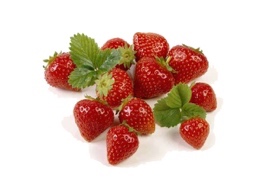** | Friendship | War |
| 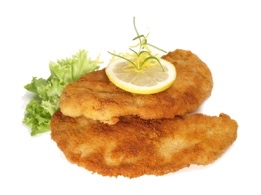 | **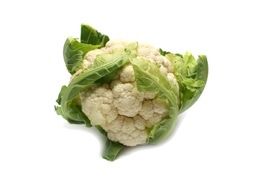** | Holiday | Murder |
| 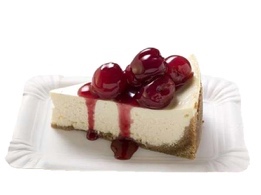 | **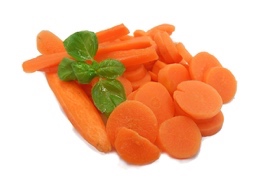** | Peace | Accident |
| 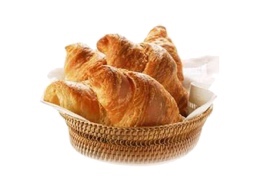 | **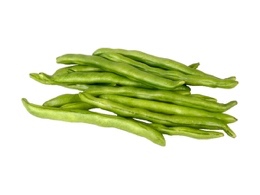** | Wonderful | Failure |
| 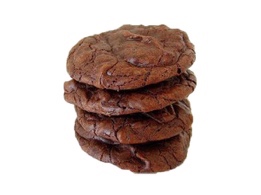 | **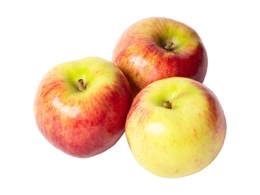** | Joy | Laziness |
| 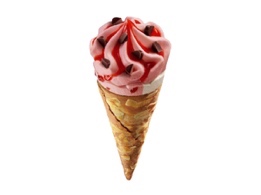 | **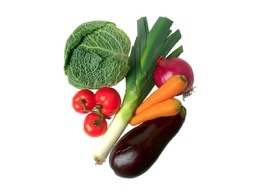** | Beauty | Disgust |
| 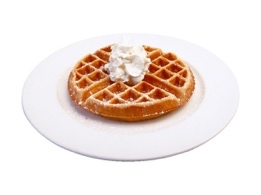 | **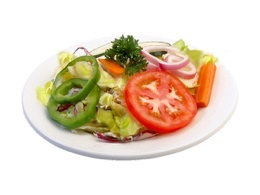** | Pleasure | Fear |

**S2.** The sequence of trial blocks in the Implicit Association Test (IAT (

| Block | Number of trials | Function | *Items allocated to the left-key response* | *Items allocated to the right-key response* |
| --- | --- | --- | --- | --- |
| 1 | 20 | Practice | Low-calorie food images | High-calorie food images |
| 2 | 20 | Practice | Unpleasant words | Pleasant words |
| 3 | 20 | Practice | Low-calorie food images  + Unpleasant words | High-calorie food images + Pleasant words |
| 4 | 40 | Test | Low-calorie food images  + Unpleasant words | High-calorie food images + Pleasant words |
| 5 | 20 | Practice | High-calorie food images | Low-calorie food images |
| 6 | 20 | Practice | High-calorie food images + Unpleasant words | Low-calorie food images + Pleasant words |
| 7 | 40 | Test | High-calorie food images + Unpleasant words | Low-calorie food images + Pleasant words |

*Note.* For half of the participants blocks 1,3 and 4 were switched with blocks 5, 6, and 7, respectively.
